# Cognitive and Academic Skills in Two Developmental Cohorts of Different Ability Level: A Mutualistic Network Perspective

**DOI:** 10.1037/h0101870

**Published:** 2021-11-08

**Authors:** Silvana Mareva, The CALM team, Joni Holmes

**Affiliations:** 1 Medical Research Council Cognition and Brain Sciences Unit, University of Cambridge

**Keywords:** learning difficulties, cognitive skills, academic skills, mutualism, network models

## Abstract

Mutualistic theories assume that the mastering of a skill, either cognitive or academic, supports and amplifies the development of other such abilities. The current study uses network science to model cross-sectional associations between cognitive and academic performance in two age-matched developmental cohorts. One cohort was a community sample drawn from the general school population, while the other included struggling learners. The community sample outperformed the struggling learners across all measures. Network models suggested that although the tasks were similarly interrelated across cohorts, there were some notable differences in association strength: Academic skills were more closely coupled in the community sample, while maths was more strongly related to cognitive skills in the struggling learners. We demonstrate the utility of network models as an analytic framework that is consistent with contemporary theories of learning difficulties and the nature of the relationship between cognitive and learning skills more broadly.

## General Audience Summary

Multiple theories attempt to explain why academic and cognitive performance are closely related. Most traditional theories assume a one-way perspective whereby specific cognitive skills constrain particular aspects of academic development. However, contemporary evidence suggests that links between cognitive skills and academic achievement are rarely specific and that the mastery of academic skills can also influence cognitive development. In this study, we use a novel statistical modelling approach consistent with the possibility that cognitive and academic skills might reciprocally influence one another, and we compare whether the same patterns of associations emerge for children drawn from the general school population and those who are struggling at school. Our modelling approach demonstrated that there are multiple direct links between cognitive and academic skills, which are broadly similar for both groups of children. Overall, we argue that researchers aiming to understand learning difficulties should consider that while cognitive skills can influence learning, the process of learning can also influence cognitive skills.

Poor academic achievement is associated with low employment rates (de Beer et al., 2014) and increased risks of mental health problems (Tempelaar et al., 2017). The need to minimise these risks and reduce associated societal and economic costs has driven decades of research that has attempted to understand and remediate learning problems. Multiple theories attempt to explain academic difficulties. The traditional, and arguably still dominant assumption, is that learning problems are caused by specific cognitive deficits (e.g., Bishop & Snowling, 2004; Melby-Lervåg et al., 2012; Pennington & Ozonoff, 1996; Szucs et al., 2013). While the unidirectional approach has generated insights that have helped guide practice and policy (e.g., Reid & Fawcett, 2008), there are several challenges that it cannot easily accommodate. More contemporary theories acknowledge that there is a more complex developmental interplay between cognitive and academic skills (e.g., Karmiloff-Smith, 2009; Peng & Kievit, 2020).

## Challenges of the Unidirectional One-to-One Mapping Perspective

Traditional theoretical models of learning difficulties have assumed that cognitive abilities provide the foundation for academic development. For example, phonological processing deficits observed among children with reading difficulties provide the basis for the hypothesis that reading difficulties are caused by poor phonological processing (Bishop & Snowling, 2004; Clayton et al., 2020; Perfetti, 2007). Similarly, working memory deficits observed among children with specific maths problems provide support for the hypothesis that memory systems are critical for maths (Szucs et al., 2013). Such theories are appealing due to the simplicity of assuming a “core deficit” (Astle & Fletcher-Watson, 2020) and their direct implications for intervention (e.g., phonological interventions for struggling readers, Bowyer-Crane et al., 2008).

Core deficit theories are challenged by evidence suggesting that similar learning difficulties may stem from different causes (i.e., equifinality). For example, reading difficulties are not wholly explained by phonological deficits. Some struggling readers have difficulties in applying letter-sound correspondences to decode words, the primary role ascribed to phonological processing in reading (Castles & Friedmann, 2014). However, other poor readers have no difficulties with decoding, but instead, struggle with reading comprehension, an aspect of reading assumed to be supported by skills such as working memory (e.g., Cain et al., 2004). Similarly, data-driven explorations of the links between cognitive and academic skills suggest that children can arrive at similar profiles of learning impairment through multiple etiological routes: Children with comparable difficulties in both reading and maths can have different cognitive profiles, such as relatively more severe problems with phonological processing or working memory (Astle et al., 2019). Consistent with equifinality, multiple deficit theories suggest that risk factors spanning multiple levels (genes, brain, cognition, and behaviour) contribute probabilistically to neurodevelopmental difficulties (Pennington, 2006) and that shared risk factors contribute to comorbidity. This idea is supported by evidence that processing speed explains some of the comorbidity between dyslexia, dyscalculia, and Attention Deficit Hyperactivity Disorder (ADHD), while language difficulties contribute to comorbidity between dyscalculia and dyslexia (McGrath et al., 2011; Peterson et al., 2017).

## Bidirectional Dynamics

A challenge to both single and multiple deficit models comes from studies showing that while cognitive skills influence academic skills, academic skills also shape cognitive development. Take our earlier example of the association between phonological processing and reading. Phonological awareness contributes to reading development, but it also benefits from reading instruction and exposure to text (Huettig et al., 2018; Nation & Hulme, 2011). Similar reciprocal effects, where the development of a given academic skill predicts growth in cognitive performance and vice-versa, have been reported between working memory and reading and maths (Miller-Cotto & Byrnes, 2020), IQ and reading (Ferrer et al., 2007, 2010), nonverbal reasoning and vocabulary (Kievit et al., 2017; Kievit, Hofman, & Nation, 2019), and executive functions and maths (Schmitt et al., 2017; Van der Ven et al., 2012). These examples suggest that difficulties in one domain may have downstream effects on other abilities and challenge the use of analytic strategies in which cognitive deficits are uniquely modelled as predictors and academic skills as outcomes.

An alternative view of the relationship between cognitive and academic skills, consistent with evidence for reciprocal benefits, is mutualism. Mutualism proposes that different abilities interact bidirectionally to reinforce one another during development (Peng & Kievit, 2020; Van Der Maas et al., 2006). In other words, the mastering of a skill supports and amplifies the development of other abilities. The mutualism model is neuro-constructivist in nature, acknowledging that specialised abilities likely emerge developmentally through a process of multidirectional interactions between genes, brain, cognition, and the environment (Karmiloff-Smith, 2009; Kievit, 2020).

In the context of cognitive-academic coupling, mutualistic transactions might be driven by experience, and particularly by educational experiences. Fundamental cognitive resources support the development of academic skills while performing academic tasks uses and trains cognitive abilities, and over time, these relationships become mutually beneficial (Peng & Kievit, 2020). For example, fluid reasoning skills aid the use of analogies and abstract schema in academic tasks, while concrete knowledge (e.g., verbal skills) supports the decomposition of complex reasoning tasks (Kievit et al., 2017). The type and strength of these transactions might be moderated by experience. Weaker or absent bidirectional relations have been reported in children with learning difficulties (e.g., Ferrer et al., 2010; Quinn et al., 2019). This could be because difficulties with specific skills operate as the bottleneck to the development of the wider cognitive system and/or because struggling learners may choose to avoid exercises that tax their area(s) of weakness. In the latter case, the net result could be that poor learners may engage in fewer activities that develop positively reinforcing associations, which over time might constrain both cognitive and academic development.

## Cognitive and Academic Skills: New Approaches

To address the challenges outlined above, it is necessary to rethink the recruitment and analysis strategies typically used to study learning difficulties. The prevailing sampling approach involves recruiting highly selective samples of children based on the presence of a specific diagnosis or difficulty, with comorbidity often treated as a confound (e.g., Szucs et al., 2013). This runs counter to a wealth of evidence showing that disorders are highly comorbid, heterogeneous, and explained by multiple causes (Astle et al., 2019; Peters & Ansari, 2019). Overstating the “purity” of learning problems at the point of recruitment biases outcomes towards core-deficit accounts. To avoid such biases, learning-related problems are increasingly studied using transdiagnostic approaches (Astle et al., 2021; Casey et al., 2014; Holmes et al., 2019; Mareva & Holmes, 2019). These approaches aim to understand the processes and causes of difficulties that occur across individuals irrespective of diagnosis or group membership.

In terms of methods, unidirectional associations between cognition and academic performance are typically modelled using latent variable approaches whereby cognitive and academic factors are derived separately, and the relations between them are mapped as directional paths from cognitive to academic skills (Peterson et al., 2017). One alternative, which can better accommodate the possibility of equifinality without assuming causational directionality, comes from network science. Network models are relatively new to cognitive science but have already proved useful in tackling similar challenges in the field of psychopathology (Borsboom, 2016). In simple terms, network models use partial correlations to depict how each observed variable relates to all other observed variables. In this way, they offer a modelling framework that is more consistent with mutualism and the possibility that reciprocal mutual transactions are the generating process behind the relationships observed across cognitive and academic skills. Applied to cross-sectional data, these models offer a tool to explore or test specific hypotheses about whether and how the complex developmental interplay between academic and cognitive skills may differ across time points and/or groups of children.

## Objectives

The aim of the current study was to apply networks models to explore and compare the interrelations between cognitive and academic abilities in a community sample and a sample of struggling learners. A heterogeneous cohort of children identified by practitioners as having school-related difficulties was included to represent struggling learners. The comparison cohort was an age-matched group of children selected as being nationally representative. For academic skills, the focus was on literacy and maths. For cognitive skills, we included assessments of processing speed, working memory, executive function, and nonverbal reasoning, all of which have been previously linked to academic performance in both typical and atypical learners (Altemeier et al., 2008; Booth et al., 2010; Gathercole et al., 2004; Geary, 2011; Green et al., 2017; Holmes et al., 2020; Mayes & Calhoun, 2007; Peng & Fuchs, 2016; Taub et al., 2008; Yeniad et al., 2013). To our knowledge, this is the first application of network science to cognitive-academic interrelationships in learners of different abilities. Consistent with mutualism and equifinality, we anticipated there would be multiple direct links between cognitive and academic skills in both cohorts. We were agnostic as to whether and how task interrelationships would differ across cohorts.

## Method

### Recruitment and Participants

Children from two cohorts were included in this study: those from the Centre for Attention, Learning, and Memory (CALM) 800 and the Nathan-Klein Institute Rockland sample (NKI-RS). These two cohorts were chosen because they include identical tasks while having different ability levels. Recruitment details, inclusion criteria, ethical and testing procedures are described in the protocols of the respective cohorts (Holmes et al., 2019; Nooner et al., 2012). Briefly, CALM includes children referred by health and educational professionals for having difficulties with attention, learning, and/or memory. Some of the children had a diagnosed learning-related problem, others had multiple diagnoses, but the majority were undiagnosed despite coming to the attention of practitioners for struggling at school. The NKI-RS is a community cohort, demographically representative of the population of Rockland, New York. Further recruitment details for each cohort are provided in Supplement Section 1.

Due to testing sessions in the NKI-RS sample sometimes being more than a year apart, we subsampled children aged 8 to 18 years-old whose assessments were completed less than six months apart from each cohort (*N*_CALM_ = 566, *N*_NKI-RS_ = 350). This time window was chosen as a liberal estimate of the child being within the same developmental period for all assessments and is consistent with previous studies using the NKI-RS cohort (e.g., Simpson-Kent et al., 2020). To ensure the comparisons were not biased by differences in sample size, CALM participants were age-matched to NKI-RS children using propensity matching based on the nearest neighbour method (Ho et al., 2011). The final age-matched samples included 350 participants (CALM: *M*_age_ = 11.26, *SD*_age_ = 2.21, 69% male; NKI-RS: *M*_age_ = 11.99, *SD*_age_ = 2.89, 56% male). As expected, children with diagnosed neurodevelopmental problems were overrepresented in CALM (based on parent report, ADHD: 32% CALM and 14% NKI-RS; Learning problems (developmental language disorder, dyslexia, dysgraphia, or dyscalculia): 11% CALM and 6% NKI-RS; Autism Spectrum Disorders (ASD): 10% CALM and 0.3% NKI-RS).

### Assessments

Assessments that were available for both cohorts were included. All tasks were taken from standardised test batteries. The psychometric properties for each assessment, together with the standardised administration procedures, can be found in the associated testing kits. Brief descriptions of each assessment are provided below.

#### Forward and Backward Digit Recall

The forward and backward digit recall tasks from the Automated Working Memory Assessment (AWMA; Alloway, 2007) were administered to CALM participants, and the same tasks from the Wechsler Intelligence Scale for Children-Revised (WISC-R; Wechsler, 1974) were administered to NKI-RS participants. In both cases, forward digit recall involved the serial recall of sequences of spoken digits, while backward digit recall required children to recall the digits in reverse serial order. The number of trials per sequence length was six for the AWMA and two for the WISC-R. The number of trials correct was scored and used in all analyses except for the permutation tests. These required identical measurement scales, so span was used to index performance for these analyses.

#### Matrix Reasoning

The Matrix Reasoning subtest of the Wechsler Abbreviated Scales of Intelligence II (WASI-II; Wechsler, 2011) was administered to children in both cohorts. Children are presented with increasingly complex nonverbal analogical reasoning problems in 2x2 matrices and are asked to select from a range of alternatives a shape that completes the pattern. The number of correctly solved matrices was scored.

#### Motor Speed

Children in both cohorts completed the Motor Speed test of the Delis Kaplan Executive Function System (D-KEFS; Delis et al., 2001). This measured time (in seconds) to trace a dotted line as quickly as possible. Completion time in seconds was scored.

#### Tower

The Tower subtest of the D-KEFS, completed by both cohorts, required children to move disks of different sizes around pegs from a start position to an end state shown on a picture following a set of rules. The number of correctly completed towers was scored.

#### Trails Number-Letter Sequencing

Both cohorts completed the Trails Number-Letter Sequencing task of the D-KEFS, which required children to connect letters and numbers in a progressive alternating sequence (e.g., 1-A, 2-B, etc). Completion time in seconds was scored.

#### Spelling, Reading, and Maths

The Spelling, Word Reading, and Numerical Operations subtests of the Wechsler Individual Achievement Test II (WIAT-II; Wechsler, 2005) were administered to children in both cohorts to measure academic abilities. Numerical operations captured number identification, counting, and the ability to solve simple and complex maths problems. Spelling required children to write down individual letters and spell single words. Word reading involved identifying letters, matching sounds to letters, and reading single words of increasing complexity. To account for the large degree of overlap in the latter two scores (see Figure S1), they were combined into a single variable called literacy.

#### Visual Scanning

The D-KEFS Visual Scanning subtest was administered to children in both cohorts. Children were required to cross out all the number threes on a response page of numbers and letters as quickly as possible. Completion time in seconds was scored.

### Analysis Plan

Network models were estimated to characterise the interrelations between cognitive and academic skills in each cohort. The accuracy of these results was scrutinised via a bootstrapping procedure. Permutation testing was then used to compare the two networks. Node strength and predictability were also estimated and compared. All statistical tests were performed in R (R Core Team, 2020, see Supplement Section 2 for library details). In all analyses, raw scores were transformed such that higher values represented better performance. For most analyses apart from node predictability estimates, missing data were handled by estimating pairwise associations. To estimate node predictability and to check the robustness of the pairwise association method, all analyses were repeated following multiple imputations via chain equations. Both approaches produced similar outcomes and are fully described in Supplement Section 3.

## Results

### Descriptive Statistics

**Figure 1 fig1:**
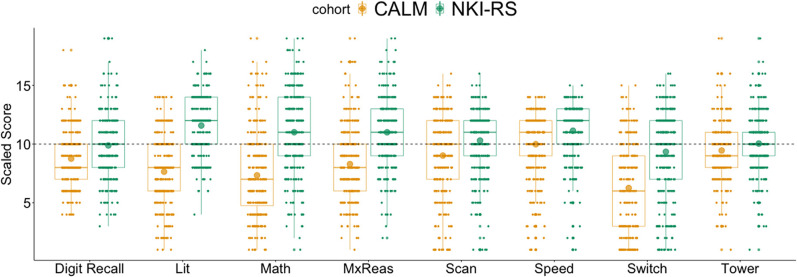
Standardised Performance Across the Tasks for CALM (Yellow, Left) and NKI-RS (Green, Right) *Note*. CALM = Centre for Attention, Learning, and Memory; NKI-RS = Nathan-Klein Institute Rockland sample. The large dots inside the boxplots show the mean in each cohort. The grey dotted line represents the age-expected mean. Norm-referenced scores were a mix of T-scores, standard scores, and scaled scores. All scores were converted to scaled scores for visualisation. Digit Recall = WISC-R/AWMA Combined forward and backward digit recall; Lit = Literacy (WIAT-II Word reading and spelling); Maths = WIAT-II Numerical operations; MxReas = WASI-II Matrix reasoning; Scan = D-KEFS Visual scanning; Speed = D-KEFS Motor speed; Switch = D-KEFS Trails number-letter sequencing task; Tower = D-KEFS Tower achievement score. See the online article for the color version of this figure.

Raw scores for each task are presented in Table 1 (for norm-referenced scores, see Figure 1). Following an adjustment for multiple comparisons, children in NKI-RS significantly outperformed CALM participants across all measures (Table S1). Pearson correlations across raw and age-regressed scores were all positive and are reported in the supplement (Figures S1 and S2). For most tasks, there were significant differences in variance between the groups (see supplement Table S3). As a robustness check, all analyses were repeated following a data transformation, which significantly reduced the differences in variance. The analyses of transformed data produced similar outcomes and are reported in the supplement (see Section 4, Table S3). [Table tbl1] [Fig fig1]

### Network Estimation and Stability

**Figure 2 fig2:**
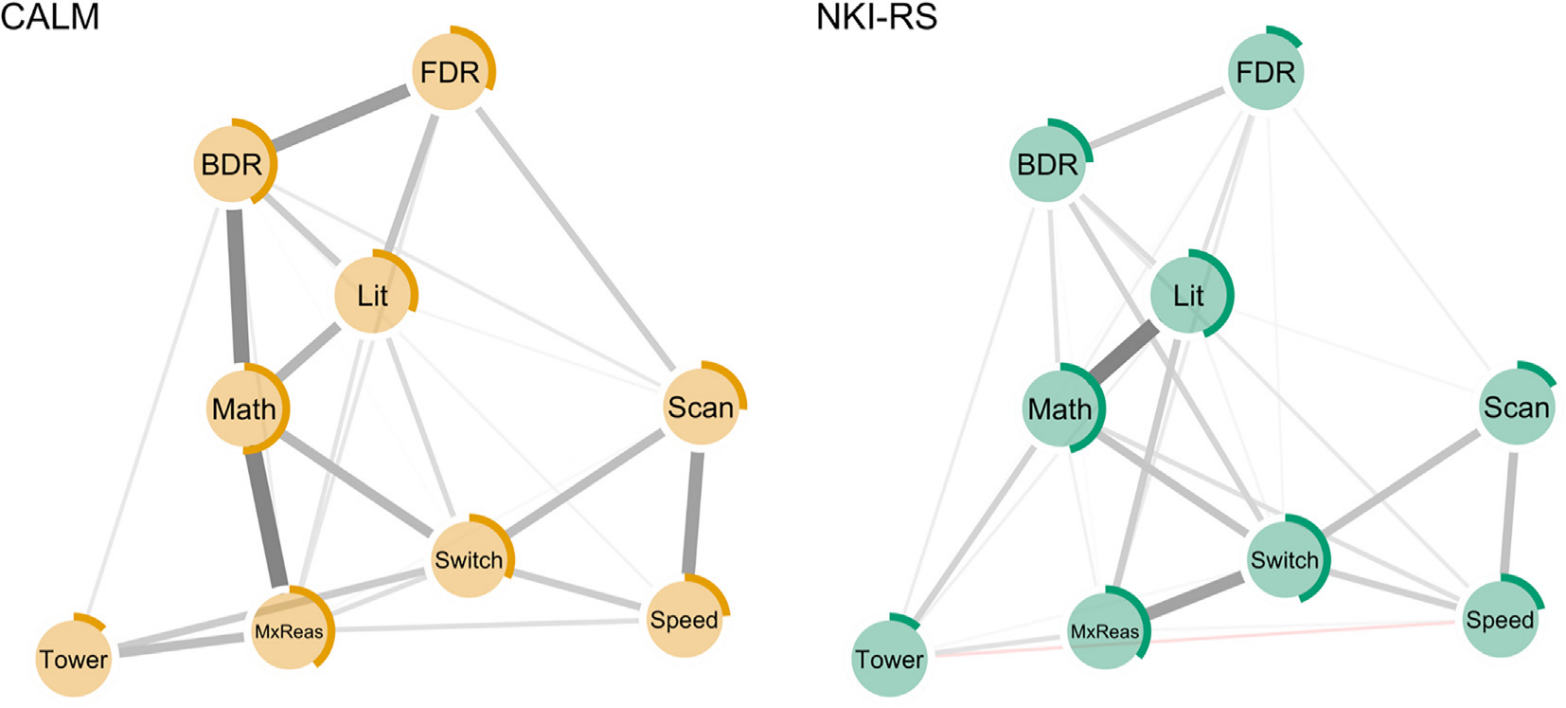
Regularised Partial Correlation Networks Across Cognitive and Academic Skills for CALM (Left) and NKI-RS (Right) *Note*. CALM = Centre for Attention, Learning, and Memory; NKI-RS = Nathan-Klein Institute Rockland sample. Age was included in the estimation but is omitted from figures. Thicker edges represent stronger associations. Red edges reflect negative associations. The rings around the nodes represent proportion of variance explained in the respective node by all connected nodes. Digit Recall = WISC-R/AWMA Combined forward and backward digit recall; Lit = Literacy (WIAT-II Word reading and spelling); Maths = WIAT-II Numerical operations; MxReas = WASI-II Matrix reasoning; Scan = D-KEFS Visual scanning; Speed = D-KEFS Motor speed; Switch = D-KEFS Trails number-letter sequencing task; Tower = D-KEFS Tower achievement score. See the online article for the color version of this figure.

A regularised partial correlation network was estimated for each cohort. In both cohorts, the analysis was underpowered to detect age moderation effects on task interrelationships of the magnitude typically reported in the literature (see supplement Section 5). Age was therefore included in the estimation but was omitted from plots and the calculation of centrality indices. In the final networks, nodes represented task performance and edge weights corresponded to the regularised partial correlation coefficient between any two tasks, controlling for age and all other scores. Each network was estimated using the graphical variant of the least absolute shrinkage and selection operator with Extended Bayesian Information Criterion used for model selection (Epskamp et al., 2018). The estimated networks are displayed in Figure 2. The bootstrapped 95% confidence intervals were small to moderate, suggesting acceptable stability (see Figures S5–11, supplement Section 6).[Fig fig2]

### Network Comparison

As a first step to comparing the networks, the correlation between edge weights across networks was estimated. The correlation was *r*_s_ = .58, indicating moderate similarity. Second, a permutation test based on 1000 iterations was used to investigate differences in network organisation (van Borkulo et al., 2017). Global connectivity was operationalised as the sum of all absolute edge weights, and global structure reflected the highest absolute difference between two corresponding edges (*M*). Permutation testing detected no significant difference in global connectivity, suggesting a similar degree of task interrelatedness in both cohorts (CALM: 4.04; NKI-RS: 4.14; *p* = .65). However, the network structure was not invariant across cohorts (*M* = 0.30, *p* < .001). Following a false discovery rate correction, four relations between tasks differed between the cohorts: Matrix reasoning – Maths: *r*_CALM_ = .34 [.25–.42], *r*_NKI-RS_ = .04 [.01–.14], *p* < .001; Literacy – Maths: *r*_CALM_ = .20 [.1–.29], *r*_NKI-RS_ = .41 [.34–.48], *p* < .001; Matrix reasoning – Switching: *r*_CALM_ = .09 [.01–.21], *r*_NKI-RS_ = .31 [.19–.40], *p* = .022; Maths – Backward digit span: *r*_CALM_ = .31 [.21–.39], *r*_NKI-RS_ = .10 [.01–.21], *p* = .027. The latter difference was not significant following the data transformation applied to address differences in task variance: *r*_CALM_ = .20, *r*_*NKI-RS*_ = .06, *p* = .27. Alternative network comparisons methods provided similar results and are reported in the supplement (see Sections 7–8, Figures S12–13, and Table S4). Finally, to compare how tasks clustered together across cohorts, a community detection algorithm was applied to each network. In both cohorts, it provided weak evidence for robust task clustering (see supplement Section 9).

### Node Centrality and Predictability

To explore and compare the relative importance of nodes within each network, node strength and predictability were estimated. Strength estimates, defined as the sum of all edge weights connected to a given node, are presented in Figure S14 (see supplement Section 10 for robustness checks). Across cohorts, node strength was strongly correlated (*r*_s_ = .75, *p* = .03) and permutation tests suggested no significant differences. Node predictability is the proportion of shared variance between a given task and all tasks related to it (Haslbeck & Waldorp, 2020). It was estimated by averaging the results of graphical models fitted to each of the 100 imputed datasets per cohort. Nodewise predictability is displayed in Figure 2. Across cohorts, average predictability estimates were similar (CALM: 32%; NKI-RS: 29%) and strongly correlated (*r*_s_ = .70, *p* = .04).

## Discussion

The current study provides one of the first demonstrations of how network approaches can be used to model the complex relationships between cognitive and academic skills proposed by contemporary developmental theories. Associations between these two domains were compared across two developmental cohorts with different levels of ability. One cohort consisted of children referred by health and educational professionals for difficulties related to learning. Their performance was significantly poorer across all tasks relative to the other cohort, which was nationally representative. Despite substantial differences in performance across cohorts, the patterns of task interrelations were broadly similar. There were multiple direct links across cognitive and academic abilities for both cohorts: The matrix reasoning, backward digit recall, and switching tasks were directly related to both literacy and maths. The presence of multiple direct links is consistent with mutualistic and multiple deficit theories (McGrath et al., 2020; Peng & Kievit, 2020) and provides evidence against specific one-to-one mappings between a single cognitive ability and a specific academic skill.

Several differences in task interrelationships were observed across cohorts. The association between maths and literacy was stronger in the community sample than in the struggling learners. Within a mutualistic perspective, education simultaneously enhances maths and literacy knowledge, and over time these become mutually enriching (Ritchie & Tucker-Drob, 2018). In struggling learners, weaker skills in one domain might slow the accumulation of knowledge and fluency, and over time, limit the mutually beneficial exchanges between domains. Additionally, negative feedback and/or experiences in one domain may lead to school disengagement or poor motivation for learning, which could have consequences beyond the affected domain (Elliot & McGregor, 2001; Wery & Thomson, 2013).

The association between maths and matrix reasoning was stronger in the struggling learners. Through a mutualistic lens, the link between maths and fluid abilities reflects both the benefits of executive abilities for learning maths and the training effects of maths practice on these cognitive abilities (Peng et al., 2019). Learning maths is executively demanding, but practice makes maths knowledge fluent and more easily available for direct retrieval (Mussolin & Noel, 2008). The stronger link in struggling learners could potentially be due to weaker maths fluency, in which case automatic solution strategies such as direct retrieval will not be available, and mathematical problem solving will instead more strongly draw on executive resources.

Finally, the relationship between the matrix reasoning and switching tasks was weaker in the poor learners. Both tasks are typically considered measures of executive abilities. However, for switching task performance to rely on higher-order cognitive control, children need to be fluent in both the alphabet and counting: Insufficient automatization of the alphabet can falsely impair performance on this task (Egeland & Follesø, 2020). Therefore, one explanation for the weaker link between tasks is the possibility that in struggling learners, poor alphanumeric knowledge constrained task performance more than executive control.

### Limitations and Future Directions

The available data constrains the inferences that can be drawn from the current study. First, we did not capture the full breadth of children’s cognitive skills and academic performance because we were limited by the measures available in both cohorts. The inclusion of broader assessments, such as reading comprehension and text-based maths problems, remains an important avenue for future work. Second, the two cohorts were drawn from different countries. Despite broad similarities in the educational systems and cultural values of these countries, it remains possible that the observed differences were due to differences in demographic or school curricula factors across cohorts. Finally, longitudinal data are needed to understand whether the differences observed across cohorts arise because certain abilities operate as a bottleneck to the development of the wider cognitive system, emerge due to different environmental or educational experiences, or a combination of the two.

This study was exploratory but demonstrates that network approaches may offer great value for the field of developmental science. They enable the simultaneous modelling of multiple routes to a specific outcome and can incorporate multidirectional interactions across levels of description. They can therefore be used to test competing theories about how abilities are related across groups and/or time points, and to characterise how genetic, neural, and environmental factors influence these relationships (e.g., Isvoranu et al., 2020; Simpson-Kent et al., 2021). Such applications will be crucial for building an understanding of not only how abilities are related but also which mechanisms enable their wiring.

### Conclusion

Using network science to model and compare the relationships between academic and cognitive skills across cohorts of children with different levels of ability, we find multiple and largely similar interrelationships, together with some key differences. These differences suggest that weak (or absent) reciprocal links between and within academic and cognitive domains may contribute to learning difficulties. Crucially, we demonstrate the potential value of network models for characterising the wiring of cognitive-academic systems across populations. Mutualistic networks provide a promising new tool for capturing complexity in development, and in time may be useful for identifying time windows where interventions could enhance mutualistic coupling.

## Supplementary Material

10.1037/h0101870.supp

## Figures and Tables

**Table 1 tbl1:** Summary Statistics for CALM and NKI-RS Based on Raw Scores, Including Number of Complete Cases and Task Abbreviations for All Assessments

Task	CALM		NKI-RS	
	*N*	*M (SD)*	*N*	*M (SD)*
Backward Digit Recall (BDR)*	348	11.44 (4.25)	301	5.28 (2.04)
Forward Digit Recall (FDR)*	349	26.38 (5.08)	301	6.97 (2.15)
Numerical Operations (Maths)	312	20.64 (8.87)	315	30.35 (10.66)
Matrix Reasoning (MxReas)	350	13.46 (5.52)	315	18.30 (4.64)
Spelling and Reading (Lit)	345	62.02 (13.18)	315	73.17 (10.81)
Visual Scanning (Scan)	301	32.13 (11.62)	338	27.62 (9.55)
Motor Speed (Speed)	299	40.14 (17.13)	338	32.44 (16.20)
Trails Number-Letter Sequencing (Switch)	270	154.31 (60.97)	337	111.67 (58.08)
Tower	279	14.44 (3.80)	339	15.46 (3.76)
*Note*. CALM = Centre for Attention, Learning, and Memory; NKI-RS = Nathan-Klein Institute Rockland sample.				
* The descriptive statistics for backward and forward digit recall reflect number of trials correct, which was used for all analyses apart from permutation tests. These requires identical measurement scales, thus, for this analysis only, span was used to index performance.
