# Supplementary material for: Cognitive and Academic Skills in Two Developmental Cohorts of Different Ability Level: A Mutualistic Network Perspective
Source: J Appl Res Mem Cogn. 2021 Nov 8;11(2):209–17. doi: 10.1037/h0101870 (PMC9254563; doi:10.1037/h0101870)
Supplement: Supplementary file 1 [file h0101870_mmc1.pdf]

Supplementary materials

Cognitive and Academic Skills in Two Developmental Cohorts of Different Ability Level: A Mutualistic Network Perspective

.

Table of Contents

Section 1: Cohort recruitment details 3
Section 2: Software and library versions 4
Table S1 5
Figure S1 6
Figure S2 7
Section 3: Results following missing data imputation with MICE 8
Table S2 9
Section 4: Homogeneity of variance 10
Table S3 11
Section 5: Investigating age effects: network moderation analyses 12
Figure S3 13
Figure S4 14
Section 6: Network stability 15
Figure S5 16
Figure S6 17
Figure S7 18
Figure S8 19
Figure S9 20
Figure S10 21
Figure S11 22
Section 7: Network comparison via confirmatory fit 23
Figure S12 24
Table S4 25
Section 8: Joint network estimation with Fused Graphical Lasso 26
Figure S13 27
Section 9: Community detection 28
Section 10: Centrality estimates 29
Figure S14 29
References 30

Section 1: Cohort recruitment details

Centre for Attention, Learning, and Memory (CALM) sample: The CALM cohort includes children referred by health and education practitioners working in the South East of England between 2014 and 2018. Referrers passed a study information pack to families of children aged 5 to 18 years who they considered to have problems with attention, learning, and/or memory. Families then contacted the CALM team if they were interested in participating. Prior to enrolment, the research team discussed the child's problems and their primary area of difficulty with referrer and the family to ensure they were eligible to take part. The only exclusion criteria applied were: 1) not being a native English speaker; 2) an uncorrected sensory impairment; 3) a confirmed genetic or neurological condition known to affect cognition. In the subsample used for the current analyses, the breakdown of referral routes was the following: 60% education (e.g. Special Educational Needs Coordinators, Educational Psychologists), 36% health (e.g. Child Psychiatrist, ADHD nurse, Paediatrician, Clinical Psychologist), 4% speech and language therapy. Additional details are available in the study protocol (Holmes et al., 2019).

Nathan-Klein Institute Rockland sample (NKI-RS): The NKI-RS cohort is a community-based lifespan sample in which key demographics (age, ethnicity, and socioeconomic status) are representative of Rockland County, New York. The country was chosen because its ethnic and economic demographics broadly resemble those of the United States. The project used zip code based recruitment (e.g., advertisements though mail and community hubs). Enrolment began in 2012 and was monitored to avoid over-representation of specific demographic groups. Later phases of the project oversampled younger and older participants to increase statistical power for ages characterised by greatest changes. Additional details are available in the study protocol (Nooner et al., 2012).

Section 2: Software and library versions

All analyses were performed in *R* (R Core Team, 2020) using the packages bootnet version 1.4.2 (Epskamp et al., 2018), factoextra version 1.0.7 (Kassambara & Mundt, 2020), igraph version 1.2.5 (Csardi & Nepusz, 2006), matchit version 3.0.2 (Ho et al., 2011), mgm version 1.2.9 (Haslbeck & Waldorp, 2015), mice version 3.9.0 (van Buuren & Groothuis-Oudshoorn, 2011), NetworkComparisonTest version 2.2.1 (Van Borkulo et al., 2016), qgraph version 1.6.5 (Epskamp et al., 2012), and psych version 1.9.12.31 (Revelle, 2019).

Table S1

Group comparisons (scaled scores) for CALM and NKI-RS.

	CALM		NKI			
	M	SD	M	SD	p-value	Cohen's d
Digit Recall	8.76	2.30	9.89	2.76	<0.001	0.45
Numerical Operations	7.34	3.61	11.01	3.52	<0.001	1.03
Matrix Reasoning	8.29	3.08	11	2.88	<0.001	0.91
Single Word Reading	7.99	3.25	11.46	2.45	<0.001	1.20
Visual Scanning	9.02	3.50	10.30	2.70	<0.001	0.41
Motor Speed	9.99	2.81	11.13	2.34	<0.001	0.45
Spelling	7.31	2.84	11.67	2.65	<0.001	1.58
Number-Letter Sequencing	6.26	3.91	9.34	3.62	<0.001	0.82
Tower Achievement	9.46	2.42	10.04	2.37	0.003	0.25

Note. Norm-referenced scores were a mix of T-scores, standard scores, and scaled scores. All scores were converted to scaled scores with a mean of 10 to facilitate interpretation. Higher scores indicate better performance. In NKI-RS, the digit recall score is based on the combined forward and backward digit recall norms provided in WISC-R. In CALM, this score reflects the average of the AWMA norm-referenced forward and backward digit recall scores. Holm-corrected p-values are shown, based on two-tailed t-tests (equal variances not assumed) and associated Cohen's d effect size estimates.

Figure S1

Pearson correlations across age, cognitive and learning skills for CALM (left) and NKI-RS (right).

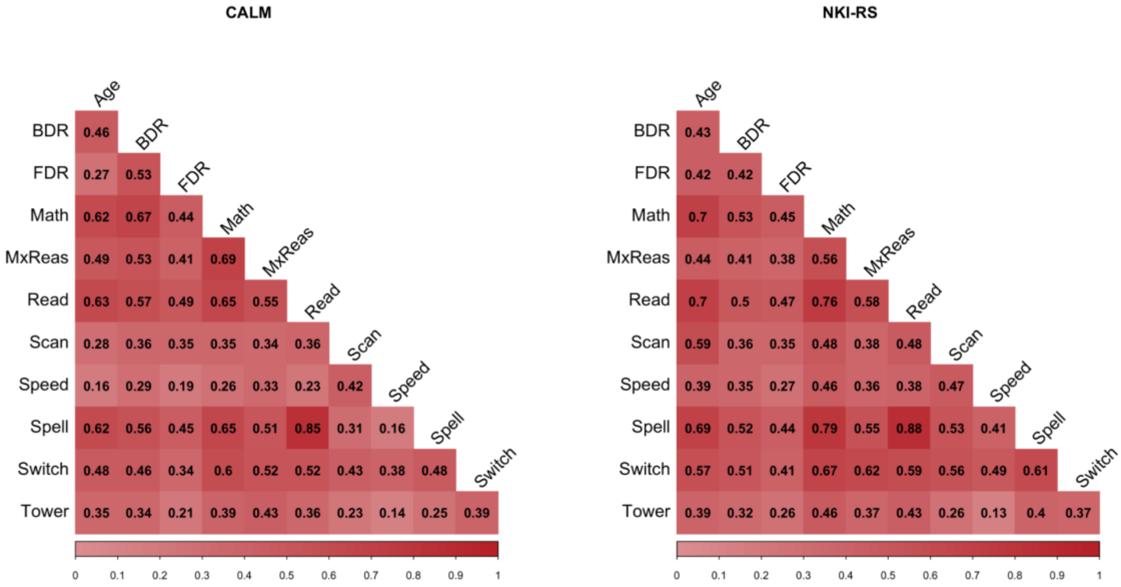

Note. BDR = AWMA/ WISC-R Backward digit recall; FDR = AWMA/ WISC-R Forward digit recall; Math = WIAT-II Numerical Operations; MxReas = WASI-II Matrix reasoning; Read = WIAT-II Word reading; Scan = D-KEFS Visual scanning; Speed = D-KEFS Motor speed; Spell = WIAT-II Spelling; Switch = D-KEFS Trails number-letter sequencing task; Tower = D-KEFS Tower.

Figure S2

Pearson correlations of age-regressed residuals across all cognitive and learning tasks for CALM (left) and NKI-RS (right).

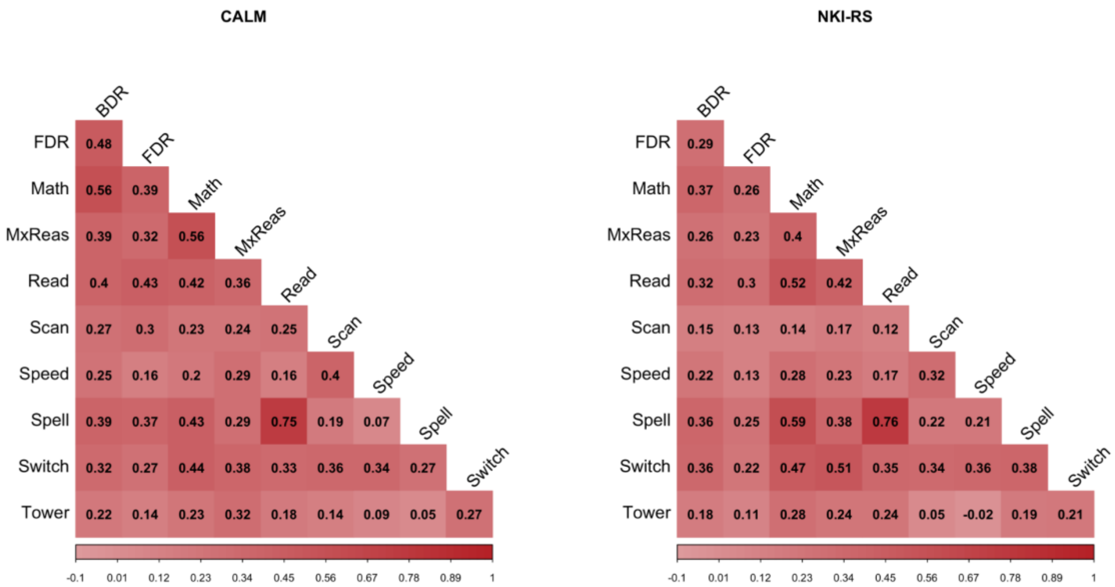

Note. BDR = AWMA/ WISC-R Backward digit recall; FDR = AWMA/ WISC-R Forward digit recall; Math = WIAT-II Numerical Operations; MxReas = WASI-II Matrix reasoning; Read = WIAT-II Word reading; Scan = D-KEFS Visual scanning; Speed = D-KEFS Motor speed; Spell = WIAT-II Spelling; Switch = D-KEFS Trails number-letter sequencing task; Tower = D-KEFS Tower.

Section 3: Results following missing data imputation with MICE

The overall proportion of missing data was similar across cohorts (NKI-RS: 8.17%; CALM: 8.74%). There is no consensus approach for handling missing data in network analysis. Therefore, for robustness, two approaches were simultaneously estimated and compared: pairwise associations and multivariate imputation by chained equations (MICE) via *R* package (van Buuren & Groothuis-Oudshoorn, 2011). The MICE procedure was used to generate 100 imputed datasets with 50 iterations per cohort, using predictive mean matching algorithms (van Buuren & Groothuis-Oudshoorn, 2011). The 100 imputed datasets per cohort were used to estimate 100 networks per cohort following the same method as described in the manuscript. The estimated task interrelations were then averaged to generate one average network model per cohort. Spearman correlation coefficients of task-interrelation estimates were calculated to examine how similar these average models were to the models presented in the manuscript (estimated with pairwise associations). The correspondence across methods was excellent: CALM – $r_s = .98$, $p < .001$; NKI-RS – $r_s = .97$, $p < .001$. Furthermore, Spearman coefficients comparing the strength centrality estimates generated based on the two approaches for handling missing data also suggested excellent agreement across methods (CALM: $r_s = 1$, $p < .001$; NKI-RS: $r_s = .98$, $p < .001$).

Finally, all cohort comparisons were repeated. The results were in agreement with those based on pairwise correlations. In terms of the similarity in task interrelationships between CALM and NKI-RS, the Spearman coefficient based on the average of the 100 imputed datasets per cohort was similar to the one presented in the manuscript ($r_s = .62$, $p < .001$). A permutation test with 1000 iterations comparing 100 pairs of imputed datasets, provided similar conclusions to those presented in the manuscript. The average observed difference in global strength was 0.10 (min = 0.003; max = 0.74), with 99% of comparisons suggesting no differences in global strength ($\alpha = 0.05$). For structure invariance, permutations based on each

of the 100 pairs of imputed datasets suggested a significant difference in global structure (α = 0.05). Looking at the edges across the 100 comparisons, the four edges flagged as significantly different between cohorts in the manuscript were the ones that most often fell below the significance threshold following a false discovery rate correction (see Table S2).

Table S2

Mean (M), Minimum (Min), and Maximum (Max) observed edge weights (ew) for CALM and NKI-RS across network estimations based on 100 imputed datasets per cohort. Mean p-values and percentage of comparisons in which edge weights were flagged as significantly different following permutation tests (adjusted for false discovery rate).

	CALM			NKI-RS				
Edge	M_{ew}	Min_{ew}	Max_{ew}	M_{ew}	Min_{ew}	Max_{ew}	$M_{p\text{-value}}$	% sign. different
Math - MxReas	0.33	0.28	0.37	0.04	0	0.10	0.0002	100
Lit - Math	0.20	0.14	0.24	0.41	0.3u	0.45	0.005	99
MxReas - Switch	0.10	0.04	0.16	0.33	0.27	0.40	0.03	83
BDR - Math	0.32	0.27	0.36	0.10	0.03	0.17	0.04	73

Note. Only edges flagged as significantly different in at least half of the comparisons are displayed in the table. BDR = WISC-R/AWMA Backward digit recall; Lit = Literacy (WIAT-II Single word reading and Spelling); Math = WIAT-II Numerical operations; MxReas = WASI-II Matrix reasoning; Switch = D-KEFS Trails number-letter sequencing task.

Section 4: Homogeneity of variance

Fligner-Killeen's non-parametric test of homogeneity of variance indicated that there was a significant difference in variance across the two cohorts (Table S3). These differences were not systematic (i.e., for some measures the variance was greater in CALM, for others the opposite was true). Differences in the variance across groups can alter the strength of the connections in the networks (Terluin et al., 2016). Therefore, to test the robustness of the results obtained from the raw data, all key analyses were repeated following a data transformation that decreased the difference in variances. The data transformation was conducted in three steps: first, the data from both cohorts was pooled together; second, participant percentage-ranks ranging from zero to one were separately estimated for each task; and finally, the resulting ranks where then mapped onto a standard normal distribution using a normal quantile function. This procedure limits the influence of univariate outliers by altering the distances between scores to resemble normal distribution, while maintaining individual ranks across tasks (Bignardi et al., 2020; Gregory, 2014). Following the transformation, the differences in variance between groups were no longer significant (Table S3). The transformed data was then analysed following the same procedures as those used with the raw data (i.e., as described in the manuscript). The networks estimated with the transformed data retained good correspondence with the networks based on the raw data (spearman correlation coefficients of task-interrelation estimates: CALM: r_s =0.96, p < .0001; NKI-RS: r_s = 0.88, p < .001). The strength centrality estimates generated based on the transformed data were also similar to the ones derived from the raw data (CALM: r_s = 0.93, p < .001; NKI-RS: r_s =0.82, p = .01). In terms of cohort comparisons, the task interrelation similarity between CALM and NKI-RS after data transformation was again moderate (r_s = 0.54, p <.001). A permutation test with 1000 iterations comparing the cohort networks, estimated following the transformation, provided similar conclusions to those suggested by the raw data. Consistent with the outcomes from the

raw data, the differences in global strength were not significant (S = 0.19, p = 0.629), and the network structures were not invariant (M = 0.31, p <0.001). Following a false discovery rate correction, three of the edges flagged as significantly different based on the analyses of the raw data were again below the significance threshold (Matrix reasoning – Maths: r_{CALM} = 0.36 , $r_{NKI\text{-}RS}$ = 0.08 , p < .001; Literacy – Maths: r_{CALM} = 0.25 , $r_{NKI\text{-}RS}$ = 0.46, p = .045; Matrix reasoning – Switching: r_{CALM} = 0.08, $r_{NKI\text{-}RS}$ = 0.29 , p = .03.The edge between Backward digit span and Maths, which was significantly different between cohorts when the raw data was analysed was not significantly different following the data transformation: r_{CALM} = 0.20 , $r_{NKI\text{-}RS}$ = 0.06 , p = .27.

Table S3

The output of Fligner-Killeen's non-parametric test of homogeneity of variance across CALM and NKI-RS before and after data transformation.

	Raw data				Transformed data			
	CALM Variance	NKI-RS Variance	χ^2	p	CALM Variance	NKI-RS Variance	χ^2	p
Backward Digit Span	0.72	1.62	17.10	<.001	0.57	0.54	0.21	0.65
Forward Digit Span	1.03	1.51	16.25	<.001	0.82	1	2.81	0.09
Literacy	179.81	116.87	13.72	<.001	0.84	0.80	0.22	0.64
Numerical Operations1	78.65	113.60	14.09	<.001	0.79	0.82	0.10	0.75
Matrix Reasoning	30.50	21.52	16.35	<.001	0.91	0.74	1.70	0.19
Visual Scanning	135.05	91.18	13.05	<.001	0.99	0.93	0.79	0.37
Motor Speed	293.35	262.53	7.94	.005	0.86	1	1.40	0.24
Number-Letter Switching	3,717.25	3,373.50	9.27	.002	0.76	0.98	3.22	0.07
Tower Achievement	14.46	14.10	0.04	0.83	0.98	0.98	0.01	0.92

Section 5: Investigating age effects: network moderation analyses

Mutualistic theories predict that the relationship between academic achievement and cognitive skills should increase with age (Peng & Kievit, 2020). This prediction is supported by meta-analyses reporting small age-related increases in the strength of the associations between cognitive and academic skills (e.g. nonverbal reasoning and reading and mathematics (Peng et al., 2019); reading and working memory (Peng et al., 2018); and executive functions and academic achievement (Jacob & Parkinson, 2015)). The sample size of the cohorts included in the current analysis was too small to investigate age effects of the size reported in meta-analyses (Perugini et al., 2018). Nonetheless, to explore this possibility a regularised Moderated Network Model (MNM) was estimated for each cohort, with age specified as a continuous moderator (Haslbeck, 2020). MNMs extend the regularised pairwise Gaussian Graphical Model to include moderation effects. The procedure was implemented in the *R* package mgm (Haslbeck & Waldorp, 2015), as follows: first, a regularised MNM with age specified as a continuous moderator was fitted to each of the hundred imputed datasets per cohort; second, the stability of the MNM results was assessed by bootstrapping (*N* boots = 100) each imputed dataset to obtain confidence intervals around the obtained estimates; finally, the bootstrapped estimates from all 100 imputed datasets were averaged for each cohort and are shown in Figure S3 and S4. Across the 100 imputed datasets per cohort age did not significantly moderate ($p \leq .05$) any of the edges in either cohort, which could likely be due to low power to detect the moderation effects typically reported in the literature.

Figure S3

The bootstrapped age moderation coefficients averaged across the 100 imputed CALM datasets are represented by the location of the dot. The width of the lines corresponds to the 95% confidence intervals. The value inside the dot corresponds to the average proportion of bootstraps in which the estimate was set as different from zero.

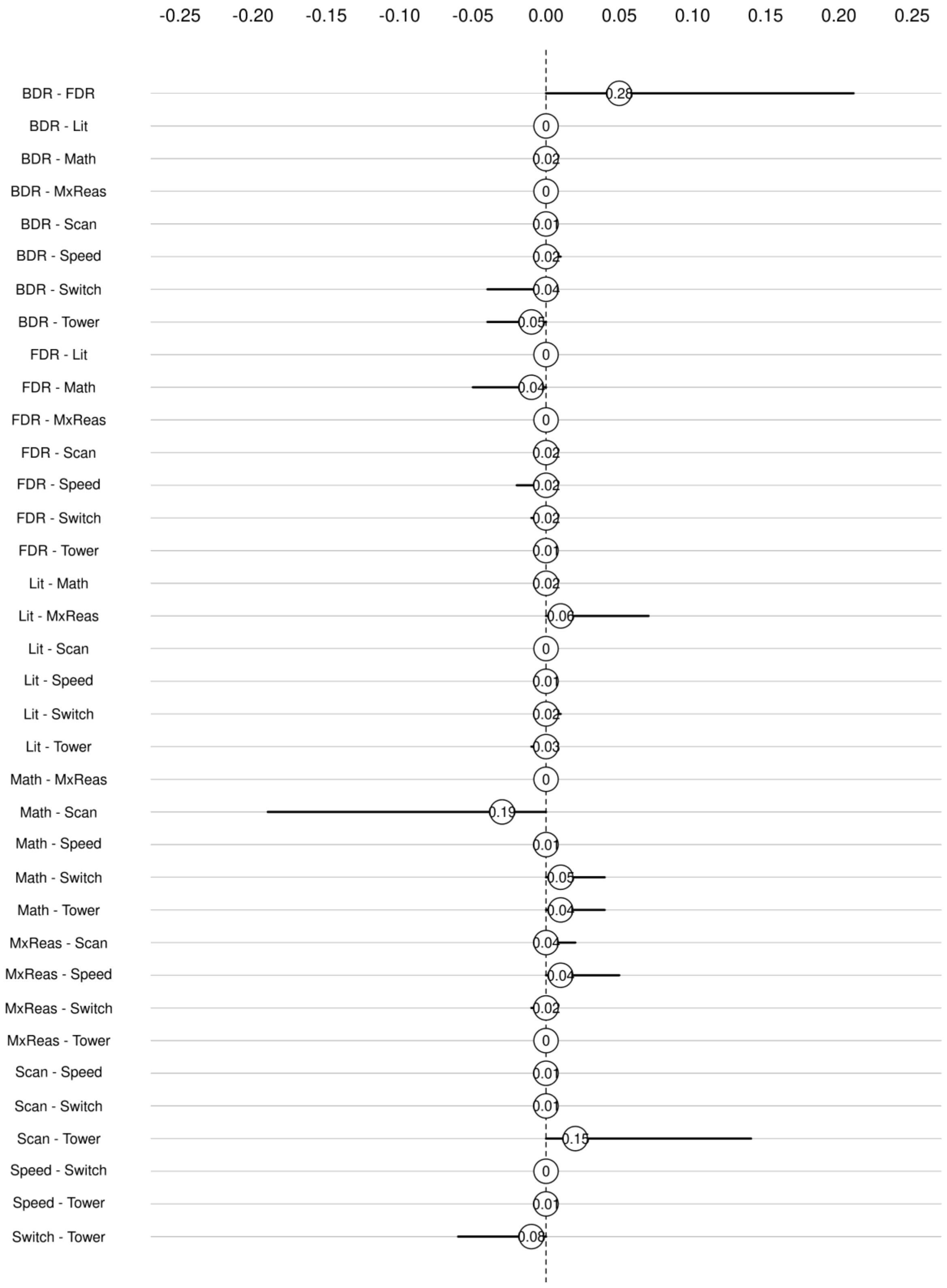

Figure S4

The bootstrapped age moderation coefficients averaged across the 100 imputed NKI datasets are represented by the location of the dot. The width of the lines corresponds to the 95% confidence intervals. The value inside the dot corresponds to the average proportion of bootstraps in which the estimate was set as different from zero.

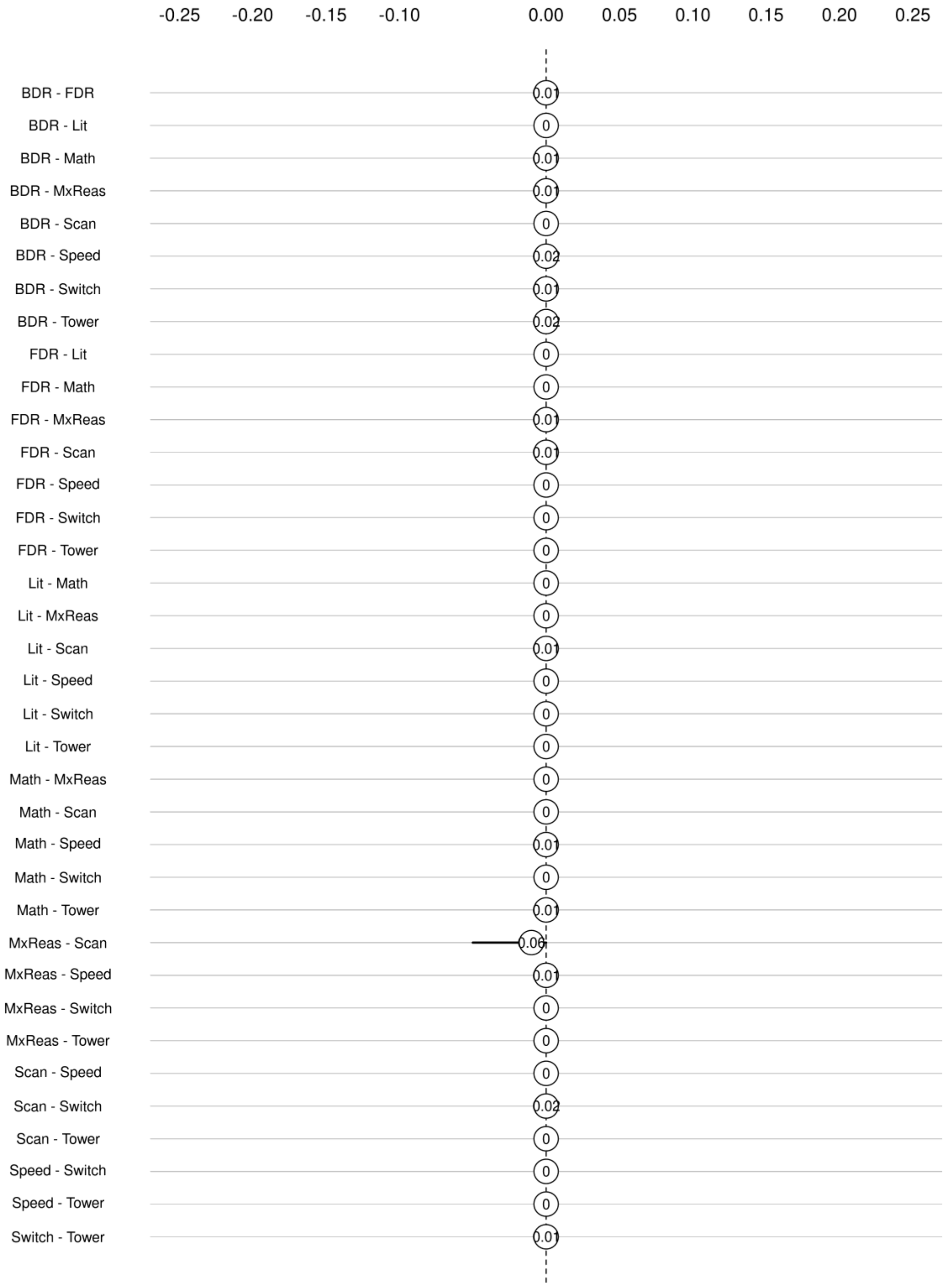

Section 6: Network stability

The bootstrapped 95% confidence intervals (CIs) around the estimated edge weights/task interrelations (N boots = 1000) are presented in Figure S5 (CALM) and Figure S6 (NKI-RS). For the most part, the widths of the bootstrapped CIs were acceptable. Notably, due to the network estimation method, the CIs are not to be interpreted as significance tests to zero (Epskamp & Fried, 2018). Instead, CIs derived from the times the parameter was not set to zero, together with the proportion of bootstrapped samples in which edges were set as different from zero across the 1000 iterations are presented in Figure S7 (CALM) and Figure S8 (NKI-RS). These results together with edge weight estimates are again summarised in Figure S9. As shown in Figure S10 and Figure S11, the smallest edge weights in each cohort had overlapping CIs and did not differ significantly from other edges, indicating that their magnitude and rank-ordering should be interpreted with caution.

Figure S5

The estimated task interrelations/edge weights for CALM are represented by the red dots and the means of the bootstrapped edge weights are represented by the black dots. The widths of the corresponding bootstrapped 95% confidence intervals indicate the edge weight accuracy.

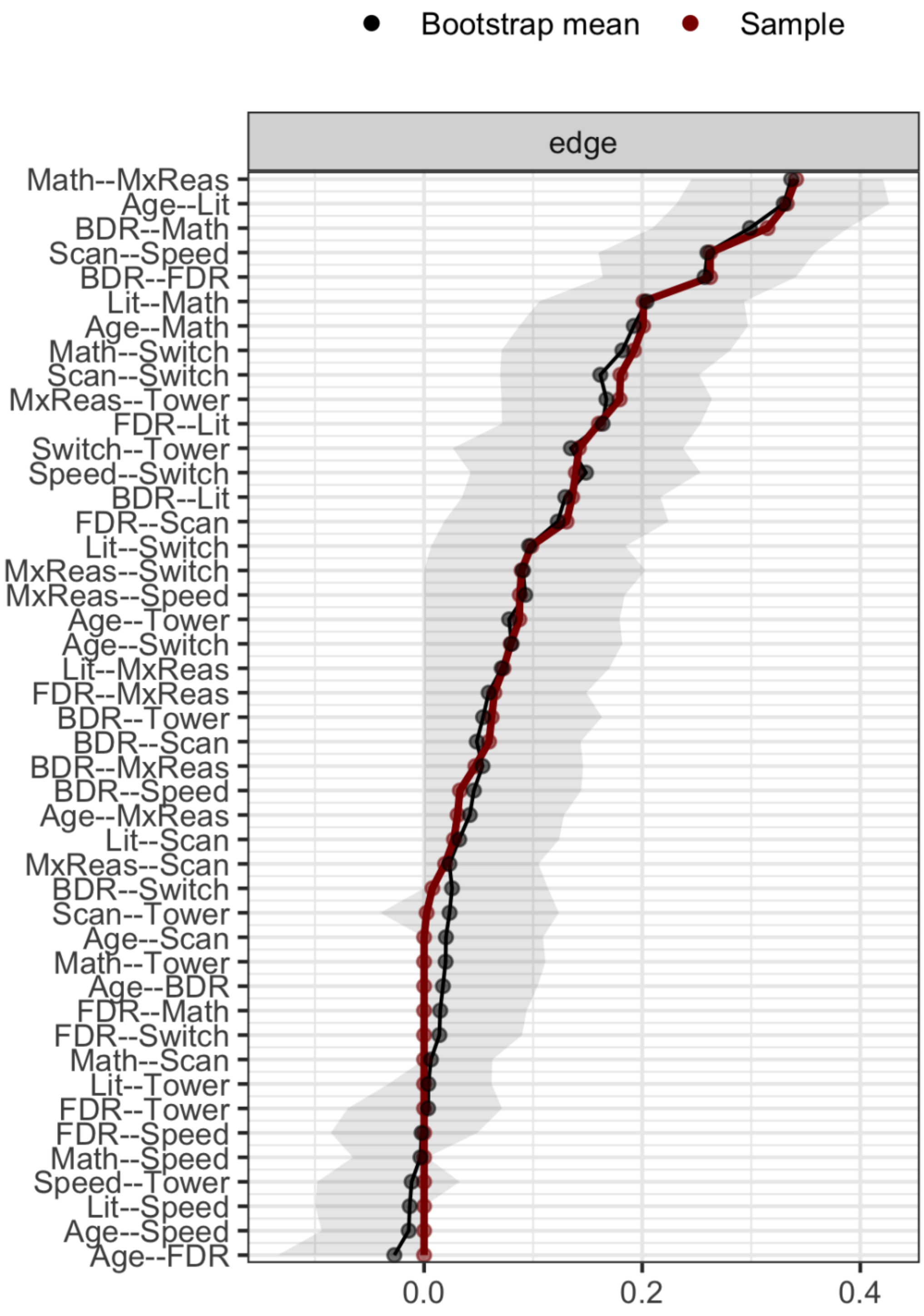

Figure S6

The estimated task interrelations/edge weights for NKI-RS are represented by the red dots and the means of the bootstrapped edge weights are represented by the black dots. The widths of the corresponding bootstrapped 95% confidence intervals indicate the edge weight accuracy.

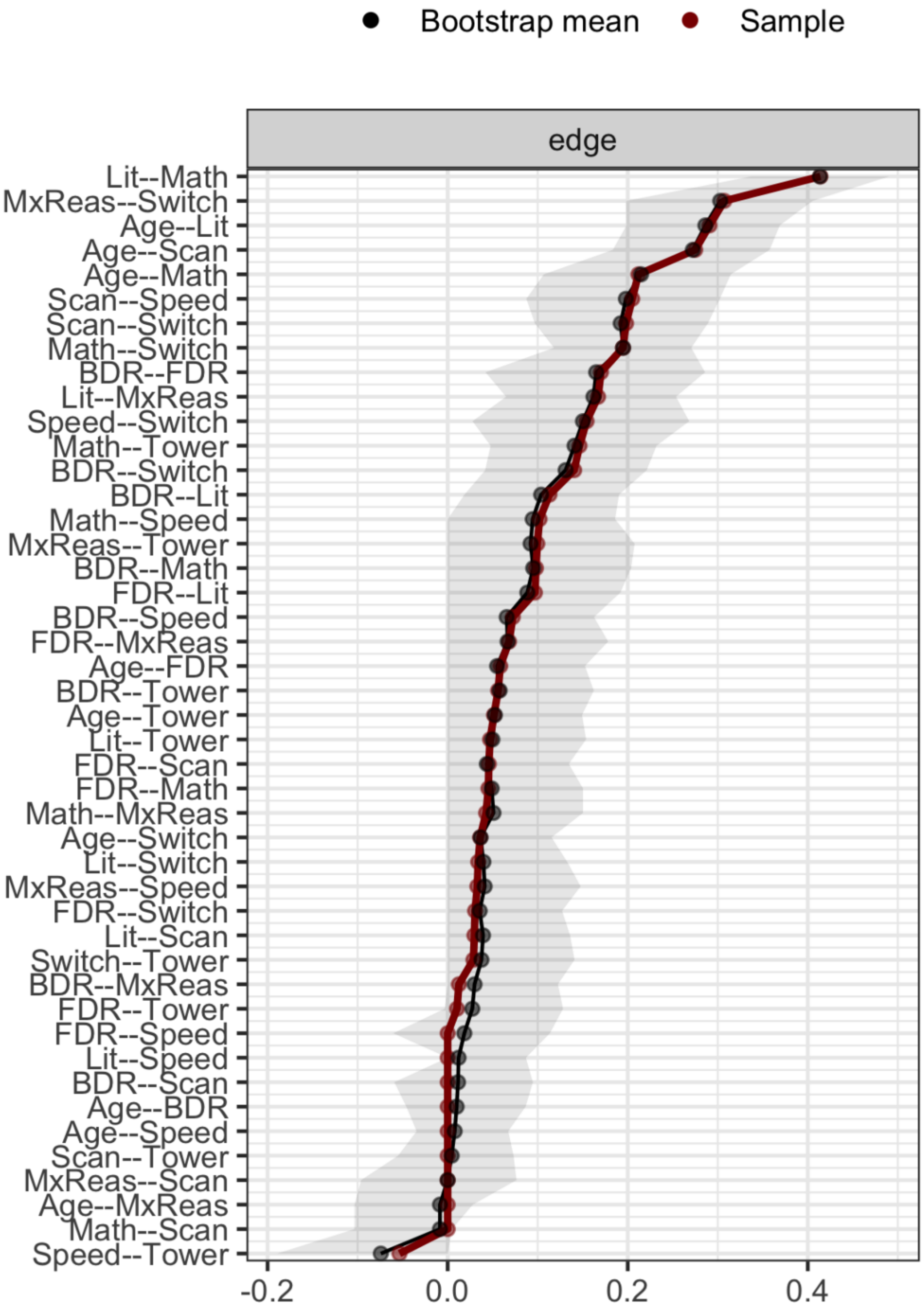

Figure S7

The estimated edge weights for CALM are represented in red and the means of the bootstrapped edge weights are in black. The width of the lines corresponds to 95% confidence intervals for the times the parameter was not set to zero. Transparency indicates how often an edge was included, with lighter lines showing that it was frequently set to zero.

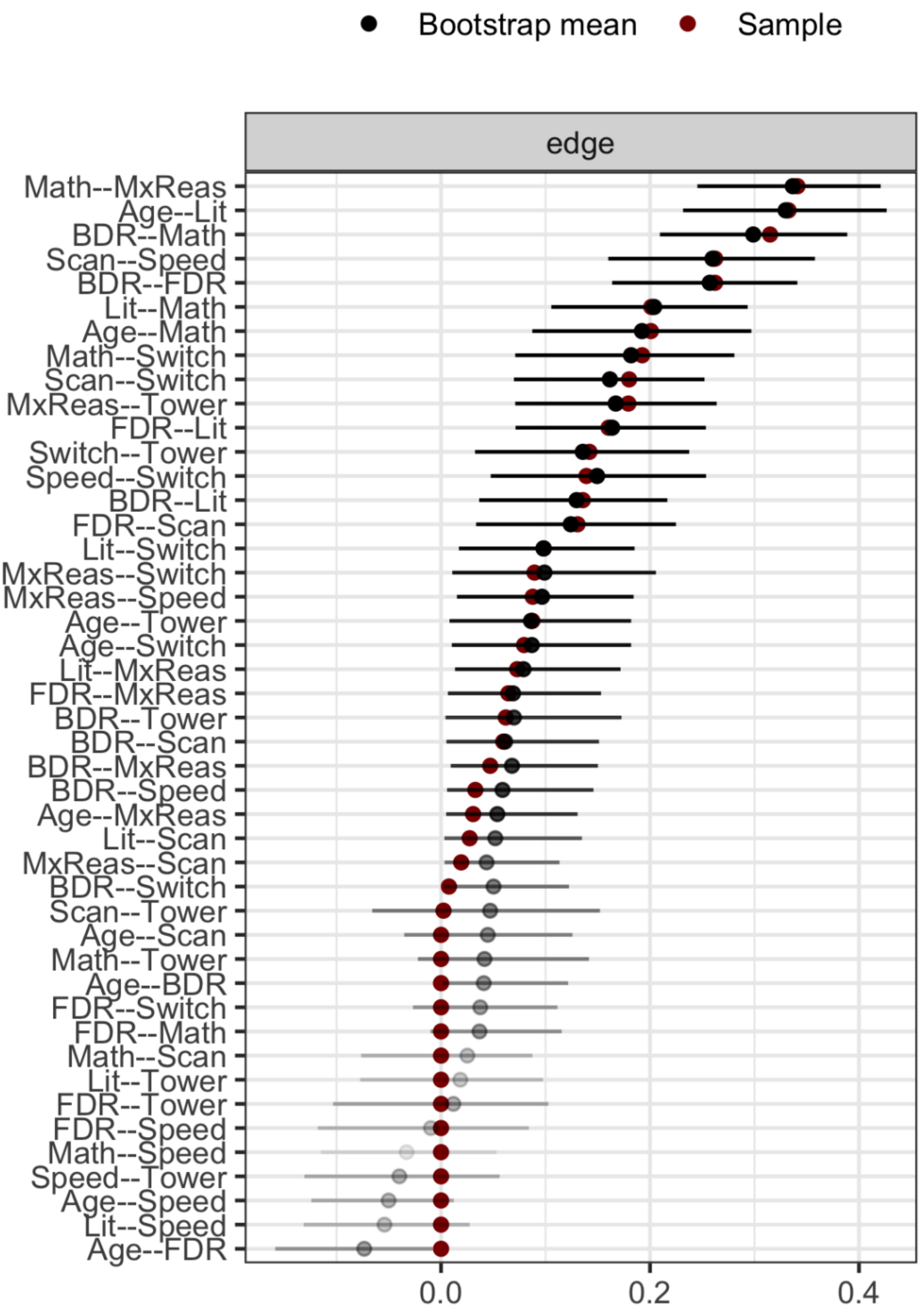

Figure S8

The estimated edge weights for NKI-RS are represented in red and the means of the bootstrapped edge weights are in black. The width of the lines corresponds to 95% confidence intervals for the times the parameter was not set to zero. Transparency indicates how often an edge was included, with lighter lines showing that it was frequently set to zero.

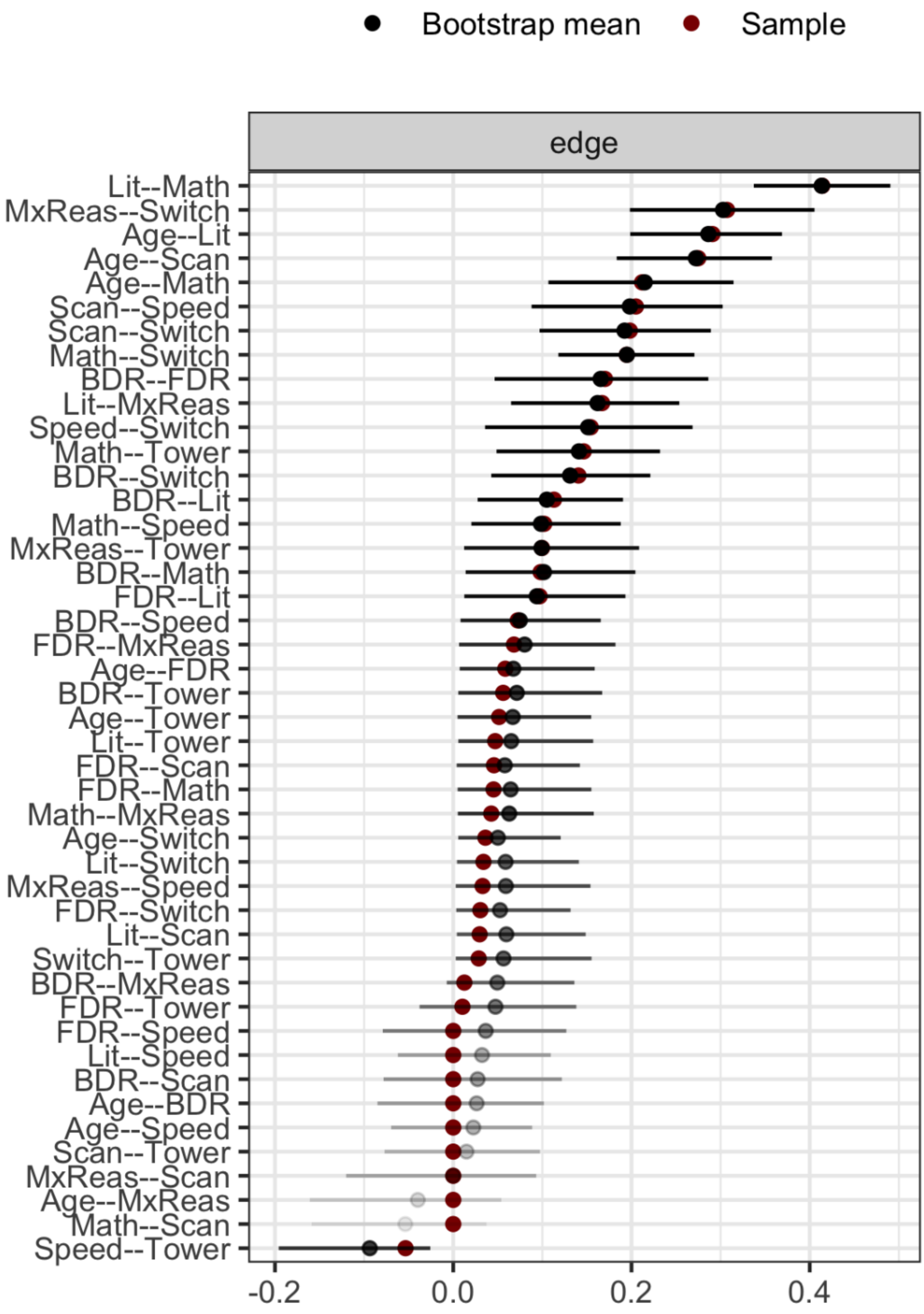

Figure S9

The upper triangle represents estimated edge weights, where darker shades correspond to stronger edge weights. The values in the lower triangle represent how often an edge was estimated to be non-zero in the 1000 bootstraps. Values of one shaded in the darkest blue indicate that an edge was included in all 1000 bootstrapped networks.

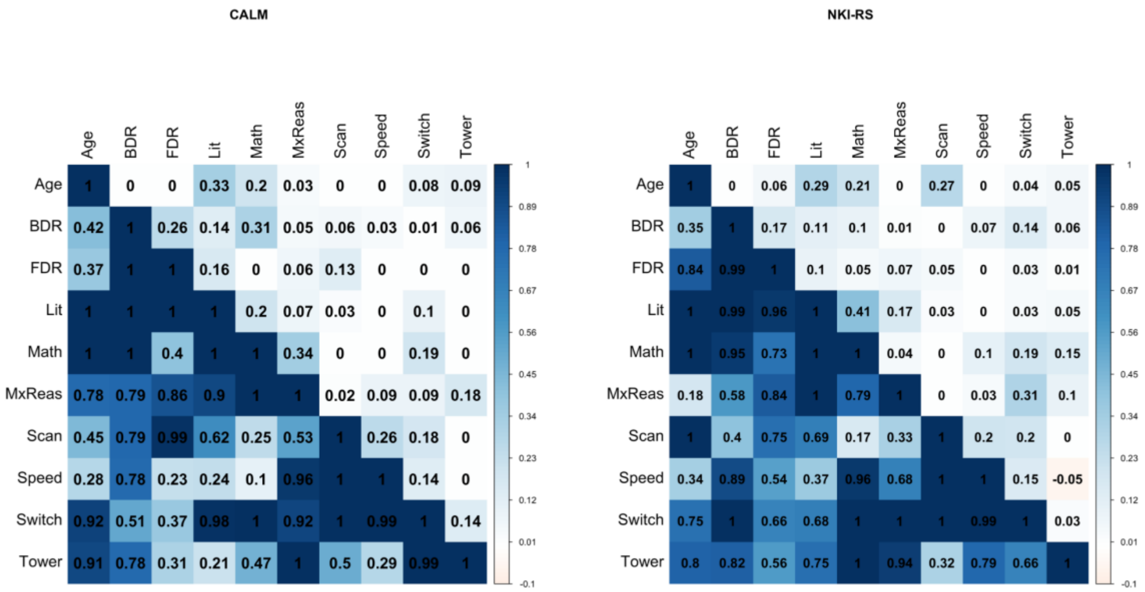

Note. BDR = WISC-R/AWMA Backward digit recall; FDR = WISC-R/AWMA Forward digit recall; Lit = Literacy (WIAT-II Single word reading and Spelling); Math = WIAT-II Numerical operations; MxReas = WASI-II Matrix reasoning; Scan = D-KEFS Visual scanning; Speed = D-KEFS Motor speed; Switch = D-KEFS Trails number-letter sequencing task; Tower = D-KEFS Tower.

Figure S10

Bootstrapped edge difference tests (α = 0.05) across non-zero task interrelations in CALM. The colours on the diagonal indicate magnitude of task interrelations, with darker colours reflecting stronger associations. The grey boxes suggest no significant difference across task interrelations, whereas the black boxes indicate significantly different task interrelations (i.e. the Math-MxReas interrelation is significantly stronger than most other edges).

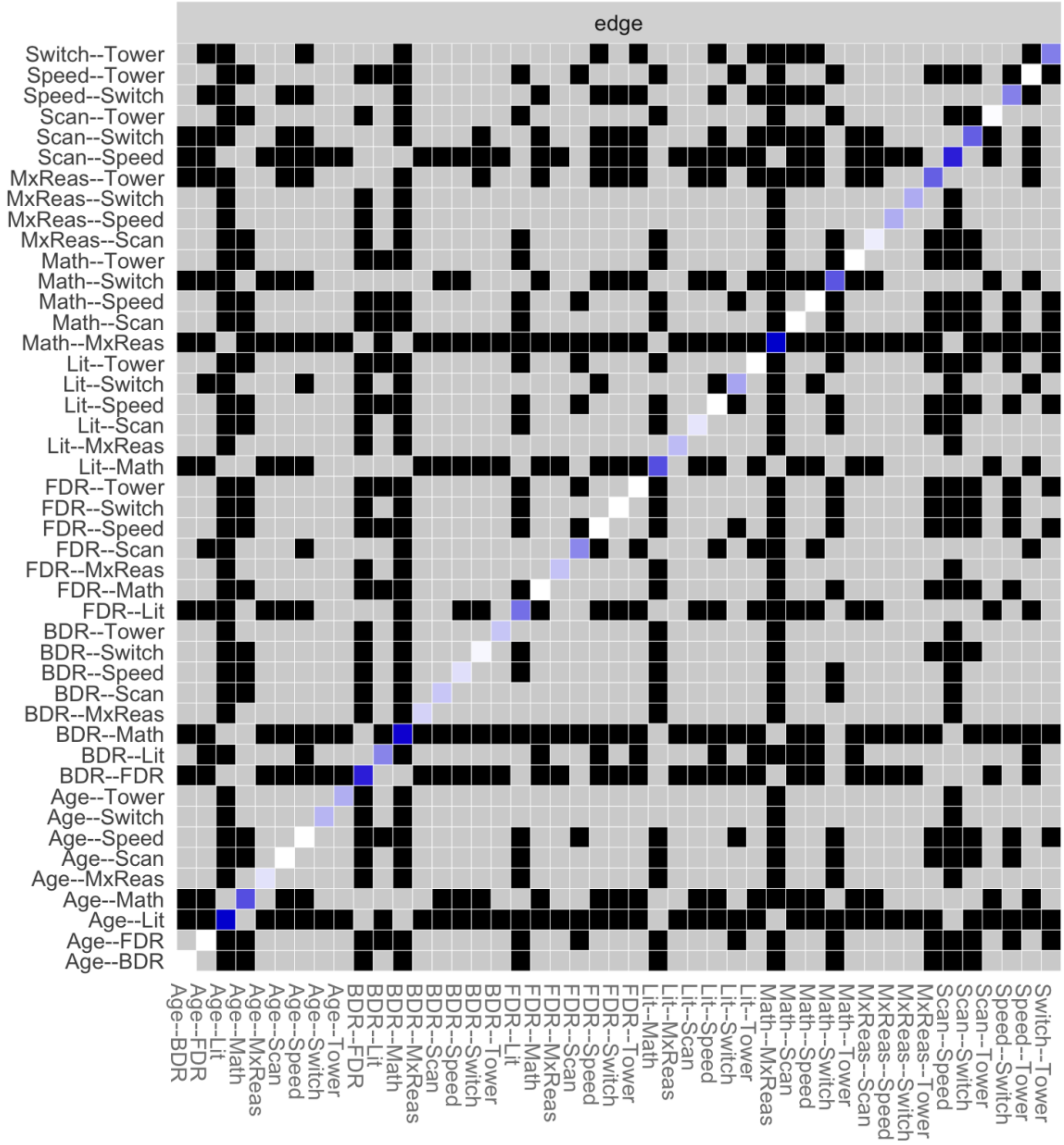

Note. BDR = WISC-R/AWMA Backward digit recall; FDR = WISC-R/AWMA Forward digit recall; Lit = Literacy (WIAT-II Single word reading and Spelling); Math = WIAT-II Numerical operations; MxReas = WASI-II Matrix reasoning; Scan = D-KEFS Visual scanning; Speed = D-KEFS Motor speed; Switch = D-KEFS Trails number-letter sequencing task; Tower = D-KEFS Tower.

Figure S11

Bootstrapped edge difference tests (α = 0.05) across non-zero task interrelations in NKI-RS. The colours on the diagonal indicate magnitude of task interrelations, with darker colours reflecting stronger associations. The grey boxes suggest no significant difference across task interrelations, whereas the black boxes indicate significantly different task interrelations (i.e. the Lit-Math interrelation is significantly stronger than most others).

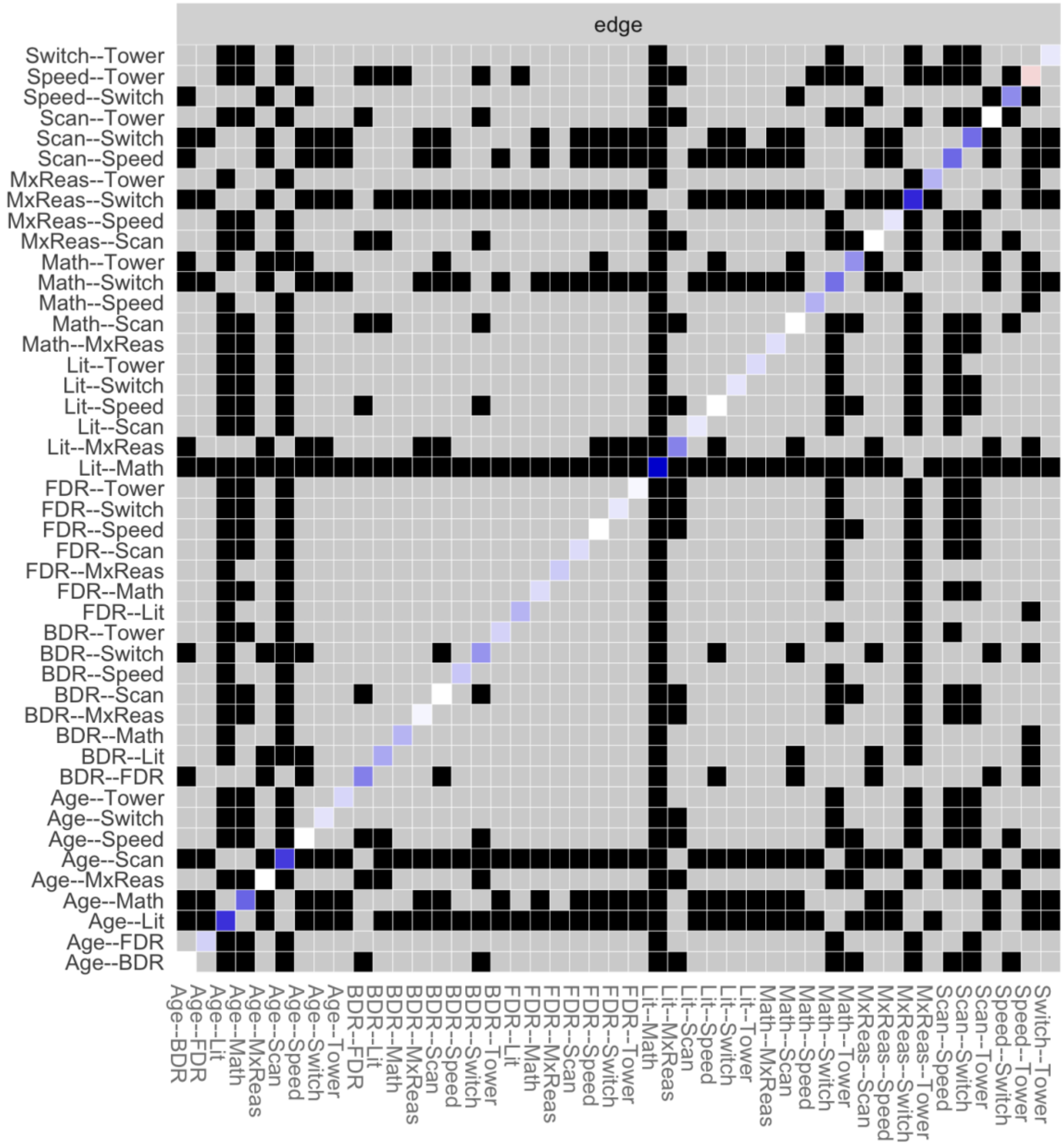

Note. BDR = WISC-R/AWMA Backward digit recall; FDR = WISC-R/AWMA Forward digit recall; Lit = Literacy (WIAT-II Single word reading and Spelling); Math = WIAT-II Numerical operations; MxReas = WASI-II Matrix reasoning; Scan = D-KEFS Visual scanning; Speed = D-KEFS Motor speed; Switch = D-KEFS Trails number-letter sequencing task; Tower = D-KEFS Tower.

Section 7: Network comparison via confirmatory fit

The robustness of the results was examined through a different analysis pipeline. For this analysis, the NKI-RS was used as a training cohort to identify the best-fitting Gaussian graphical model via the R package psychonetrics, version 0.7.2 (Epskamp, 2020). The fit of the identified model was then tested in CALM. In the training cohort (NKI-RS), the prune function was used to automatically and reclusively remove parameters which do not meet the $\alpha = 0.05$ level. This was done in combination with the stepup function, which adds edges according to modification indices at $\alpha = 0.05$ level. This approach resulted in a sparser network compared to the regularisation procedure presented in the paper (see the resulting network in Figure S12). Subsequently, the model structure identified in NKI-RS (i.e. the edges included in the final NKI-RS model) was then fitted to CALM. Fit indices suggested poor fit (see Table S4). Modification indices were then examined to flag potential differences in the task interrelation structure. In agreement with the results of the permutation testing reported in the manuscript, the top modification index pointed at one of the edges, which was identified as significantly different based on the permutation test: Maths - Matrix Reasoning. Allowing this parameter to be included in the CALM model significantly improved the fit (see Table S4). For the remaining estimates, the largest differences in edge weights based on this analysis pipeline were consistent with those reported in the paper (Maths – Literacy, Maths – Backward digit recall, Matrix reasoning – Switching).

Figure S12

Displayed on the right are model parameters for the model identified based on NKI-RS data. Shown on the left are parameters estimated following a confirmatory fit of the same model structure to the CALM data with one modification (Maths – Matrix Reasoning).

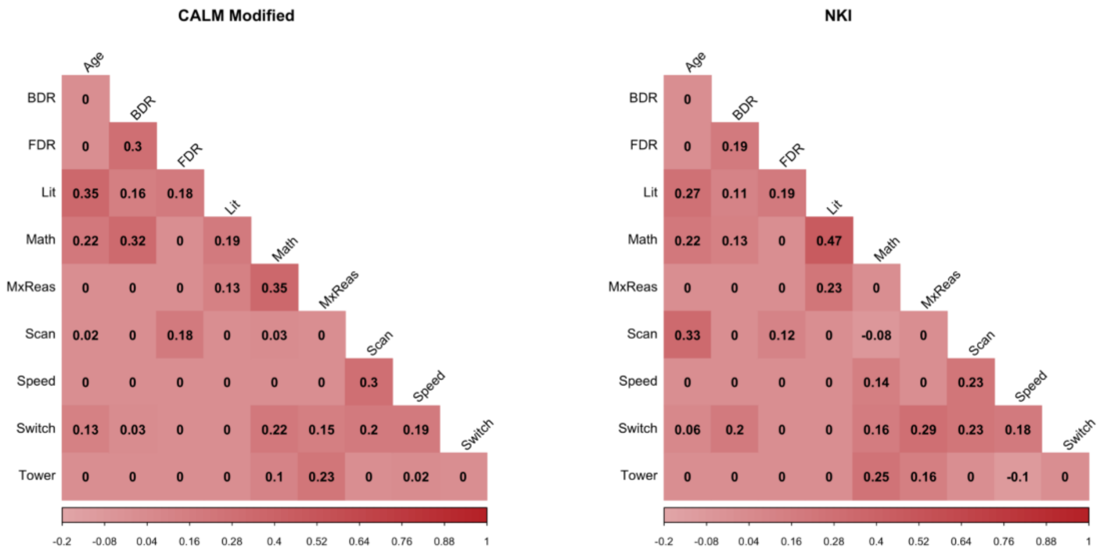

Note. BDR = WISC-R/AWMA Backward digit recall; FDR = WISC-R/AWMA Forward digit recall; Lit = Literacy (WIAT-II Single word reading and Spelling); Math = WIAT-II Numerical operations; MxReas = WASI-II Matrix reasoning; Scan = D-KEFS Visual scanning; Speed = D-KEFS Motor speed; Switch = D-KEFS Trails number-letter sequencing task; Tower = D-KEFS Tower.

Table S4

Model fit indices following model identification based on NKI-RS data, confirmatory fit of NKI-RS identified structure on CALM data, and a modified model for CALM based on modification indices (i.e. including the edge Maths – Matrix Reasoning).

	χ^2	DF	p	BIC	CFI	TLI	RMSEA	Δ χ^2	Δ DF	Δ p
NKI-RS	28.52	23	0.20	20,269	1	0.99	0.03			
CALM	116.07	23	0	21,694	0.94	0.88	0.11			
CALM Mod.	55.25	22	0	21,639	0.98	0.95	0.07	60.82	1	< .001

Note. BIC = Bayesian Information Criterion; CFI = Comparative Fit Index; TLI = Tucker Lewis index; RMSEA = Root Mean Square Error of Approximation.

Section 8: Joint network estimation with Fused Graphical Lasso

Joint network estimation via fused graphical lasso (FGL) provides an alternative approach for investigating patterns of similarities and differences across groups (Danaher et al., 2014). The main advantage of FGL over other methods is that it exploits the similarities across groups to improve estimates, without masking differences across groups (Costantini et al., 2019). The method has previously been employed to compare symptom interrelations across individuals with diagnosed psychiatric conditions and community samples (Richetin et al., 2017). Similar to the graphical least absolute shrinkage and selection operator, the FGL uses a tuning parameter to apply a penalty to the sum of absolute values of the concentration matrix elements. In addition, it uses a second tuning parameter to penalise the sum of absolute values of the differences between the corresponding concentration matrix elements across groups. In the current analysis, the values of both tuning parameters were selected based on information criteria (EBIC) for consistency with the analysis presented in the manuscript. All analysis were implemented in the *R* package EstimateGroupNetwork, version 0.2.2 (Costantini & Epskamp, 2017). The resulting estimates are displayed in Figure S13. Overall, the majority of edge weights were set as different and the results retained substantial similarity to those based on the networks presented in the manuscript (CALM: $r_s = 1$, $p <.001$; NKI-RS: $r_s = 0.99$, $p <.001$). The degree of similarity across cohorts was again moderate – $r_s = .65$, $p < .001$. The largest differences in cohort edge weights corresponded to those identified through the separate model estimation presented in the manuscript: Maths – Matrix Reasoning, Maths – Backward Digit Recall, Maths – Literacy, and Maths – Switching.

Figure S13

Edge weights for CALM and NKI-RS following a joint network estimation based on fused graphical lasso.

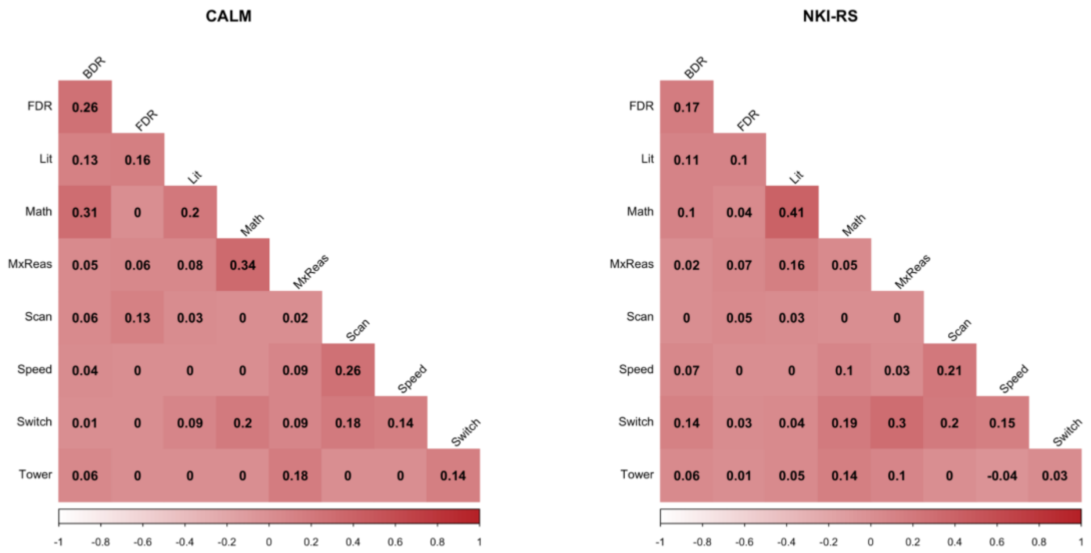

Note. BDR = WISC-R/AWMA Backward digit recall; FDR = WISC-R/AWMA Forward digit recall; Lit = Literacy (WIAT-II Single word reading and Spelling); Math = WIAT-II Numerical operations; MxReas = WASI-II Matrix reasoning; Scan = D-KEFS Visual scanning; Speed = D-KEFS Motor speed; Switch = D-KEFS Trails number-letter sequencing task; Tower = D-KEFS Tower.

Section 9: Community detection

The Walktrap algorithm (Pons & Latapy, 2006) was applied to each network to evaluate the extent to which tasks could be grouped together in each cohort. The algorithm searches for densely connected parts of the network by taking recursive random walks across pairs of nodes. The modularity index (Q) was used to assess the quality of the partition, with higher values indicating stronger partitions and values around 0.3 considered evidence for weakly defined communities (Newman & Girvan, 2004). In both cohorts, the index was substantially below 0.3 (Q_{CALM} = 0.15 and Q_{NKI-RS} = 0.08), indicating the tasks did not form robust groups.

Section 10: Centrality estimates

Strength centrality derived from the networks presented in the manuscript are displayed in Figure S14. Case-dropping analysis was used to assess the stability of these estimates: the metric was considered stable if at least half of the children within each sample could be dropped while still retaining 95% probability of a 0.7 correlation between the strength estimates based on the full sample and those derived from subsamples (Epskamp & Fried, 2018), these recommendation was met in both samples: max. drop proportion CALM: 67% and NKI-RS: 75%. Finally, to check whether node strength was influenced by differential variability across tasks (Terluin et al., 2016), Spearman correlation coefficients were calculated across node strength and the standard deviation for each task. They were weak to moderate and not statistically significant: CALM: r_s =,0.08 p = 0.84; NKI-RS: r_s = 32, p = 0.41.

Figure S14

Centrality estimate strength across CALM and NKI-RS.

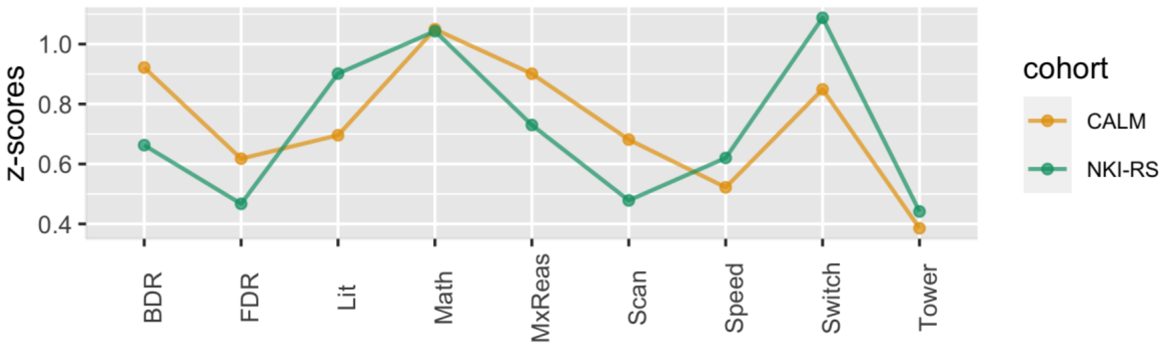

Note. BDR = WISC-R/AWMA Backward digit recall; FDR = WISC-R/AWMA Forward digit recall; Lit = Literacy (WIAT-II Single word reading and Spelling); Math = WIAT-II Numerical operations; MxReas = WASI-II Matrix reasoning; Scan = D-KEFS Visual scanning; Speed = D-KEFS Motor speed; Switch = D-KEFS Trails number-letter sequencing task; Tower = D-KEFS Tower.

References

Bignardi, G., Dalmaijer, E., Anwyl-Irvine, A. L., & Astle, D. (2020). *Collecting Big Data with Small Screens: Group-Tests of Children's Cognition with Touchscreen Tablets are Reliable and Valid*. https://doi.org/10.31219/osf.io/aw6c5

Costantini, G., & Epskamp, S. (2017). *Package ' EstimateGroupNetwork .'* https://mran.revolutionanalytics.com/snapshot/2017-04-01/web/packages/EstimateGroupNetwork/EstimateGroupNetwork.pdf

Costantini, G., Richetin, J., Preti, E., Casini, E., Epskamp, S., & Perugini, M. (2017). Stability and variability of personality networks. A tutorial on recent developments in network psychometrics. *Personality and Individual Differences*, *136*, 68–78. https://doi.org/10.1016/j.paid.2017.06.011

Csardi, G., & Nepusz, T. (2006). The igraph software package for complex network research. *International Journal of Complex Systems*. http://www.necsi.edu/events/iccs6/papers/c1602a3c126ba822d0bc4293371c.pdf

Danaher, P., Wang, P., & Witten, D. M. (2014). The joint graphical lasso for inverse covariance estimation across multiple classes. *Journal of the Royal Statistical Society: Series B (Statistical Methodology)*, *76*(2), 373–397. https://doi.org/10.1111/rssb.12033

Epskamp, S. (2020). *psychonetrics: Structural Equation Modeling and Confirmatory Network Analysis* (version 0.6.). Article version 0.6. https://cran.r-project.org/package=psychonetrics

Epskamp, S., Borsboom, D., & Fried, E. I. (2018). Estimating psychological networks and their accuracy: A tutorial paper. *Behavior Research Methods*, *50*(1), 195–212. https://doi.org/10.3758/s13428-017-0862-1

Epskamp, S., Cramer, A. O. J., Waldorp, L. J., Schmittmann, V. D., & Borsboom, D. (2012). **qgraph** : Network Visualizations of Relationships in Psychometric Data. *Journal of Statistical Software*, *48*(4). https://doi.org/10.18637/jss.v048.i04

Epskamp, S., & Fried, E. I. (2018). A Tutorial on Regularized Partial Correlation Networks. *Psychological Methods*, *23*(4), 617–634. https://doi.org/10.1037/met0000167

Gregory, R. (2014). *Psychological Testing: History, Principles, and Applications* (7th ed.). Pearson Education.

Haslbeck, J. (2020). *Estimating Group Differences in Network Models using Moderation Analysis*. https://doi.org/10.31234/osf.io/926pv

Haslbeck, J., & Waldorp, L. (2015). MGM: Estimating Time-Varying Mixed Graphical Models in High-Dimensional Data. In *JSS Journal of Statistical Software*. http://arxiv.org/abs/1510.06871

Ho, D. E., Imai, K., King, G., & Stuart, E. A. (2011). MatchIt: Nonparametric preprocessing for parametric causal inference. *Journal of Statistical Software*, *42*, 1–28. http://www.jstatsoft.org/

Holmes, J., Bryant, A., & Gathercole, S. E. (2019). Protocol for a transdiagnostic study of children with problems of attention, learning and memory (CALM). *BMC Pediatrics*, *19*(1), 10. https://doi.org/10.1186/s12887-018-1385-3

Jacob, R., & Parkinson, J. (2015). The Potential for School-Based Interventions That Target Executive Function to Improve Academic Achievement: A Review. *Review of Educational Research*, *85*(4), 512–552. https://doi.org/10.3102/0034654314561338

Kassambara, A., & Mundt, F. (2020). *factoextra: Extract and Visualize the Results of Multivariate Data Analyses*. https://cran.r-project.org/web/packages/factoextra/factoextra.pdf

Nooner, K. B., Colcombe, S. J., Tobe, R. H., Mennes, M., Benedict, M. M., Moreno, A. L., Panek, L. J., Brown, S., Zavitz, S. T., Li, Q., Sikka, S., Gutman, D., Bangaru, S., Schlachter, R. T., Kamiel, S. M., Anwar, A. R., Hinz, C. M., Kaplan, M. S., Rachlin, A. B., … Milham, M. P. (2012). The NKI-Rockland Sample: A Model for Accelerating the Pace of Discovery Science in Psychiatry. *Frontiers in Neuroscience*, *6*, 152. https://doi.org/10.3389/fnins.2012.00152

Peng, P., Barnes, M., Wang, C. C., Wang, W., Li, S., Swanson, H. L., Dardick, W., & Tao, S. (2018). Meta-analysis on the relation between reading and working memory. *Psychological Bulletin*, *144*(1), 48–76. https://doi.org/10.1037/bul0000124

Peng, P., & Kievit, R. A. (2020). The Development of Academic Achievement and Cognitive Abilities: A Bidirectional Perspective. *Child Development Perspectives*, *14*(1), 15–20. https://doi.org/10.1111/cdep.12352

Peng, P., Wang, T., Wang, C. C., & Lin, X. (2019). A meta-analysis on the relation between fluid intelligence and reading/ mathematics: Effects of tasks, age, and social economics status. *Psychological Bulletin*, *145*(2), 189–236. https://doi.org/10.1037/bul0000182

Perugini, M., Gallucci, M., & Costantini, G. (2018). A practical primer to power analysis for simple experimental designs. *International Review of Social Psychology, 31*(1), 1–23. https://doi.org/10.5334/IRSP.181

R Core Team. (2020). *R: A language and environment for statistical computing.* (p. https://www.R-project.org/). https://www.r-project.org/

Revelle, W. (2019). *psych: Procedures for Psychological, Psychometric, and Personality Research* (1.9.12). https://cran.r-project.org/package=psych

Richetin, J., Preti, E., Costantini, G., & De Panfilis, C. (2017). *The centrality of affective instability and identity in Borderline Personality Disorder: Evidence from network analysis*. https://doi.org/10.1371/journal.pone.0186695

Terluin, B., de Boer, M. R., & de Vet, H. C. W. W. (2016). Differences in Connection Strength between Mental Symptoms Might Be Explained by Differences in Variance: Reanalysis of Network Data Did Not Confirm Staging. *PLoS ONE, 11*(11), e0155205. https://doi.org/10.1371/journal.pone.0155205

Van Borkulo, C. D., Epskamp, S., & Maintainer, A. M. (2016). *Statistical Comparison of Two Networks Based on Three Invariance Measures*. https://cran.r-project.org/web/packages/NetworkComparisonTest/NetworkComparisonTest.pdf

van Buuren, S., & Groothuis-Oudshoorn, K. (2011). mice: Multivariate Imputation by Chained Equations. *Journal of Statistical Software, 45*(3), 1–67.
